# A novel genotyping technique for discriminating LVAS-associated high-frequency variants in *SLC26A4* gene

**DOI:** 10.1186/s13568-020-01102-7

**Published:** 2020-09-15

**Authors:** Chen Zhou, Xiangman Zou, Cuiying Peng, Guoqiang Gao, Zifen Guo

**Affiliations:** 1 Institute of Pharmacy and Pharmacology, University of South China, Hengyang, Hunan China; 2 Hunan Province Cooperative Innovation Center for Molecular Target New Drug Study, Hengyang, Hunan China; 3 The Second Affiliated Hospital, University of South China, Hengyang, Hunan China

**Keywords:** Solute carrier family 26 member 4, Large vestibular aqueduct syndrome, Exo^+^ polymerase, Phosphorothioate modification

## Abstract

An increasing number of biological and epidemiological evidence suggests that c.919-2A > G and c.2168A > G variants of solute carrier family 26, member 4 (*SLC26A4*) gene play a critical role in the development of large vestibular aqueduct syndrome (LVAS). In this study, we developed a rapid genotyping method for discriminating LVAS-associated high-frequency variants in *SLC26A4* gene. The genotyping technique consists of 3′ terminal exonuclease-resistant phosphorothioate-modified allele specific primer extension mediated by exo^+^ polymerase. In PCR amplification by Pfu polymerase, allelic specific primers perfectly matching wild type allele were extended while no specific products were yielded from primers targeting variant allele. Similarly, allelic specific primers perfectly matching variant allele were extended and no specific products were observed from primers targeting wild type allele. The clinical application of 3′ terminal phosphorothioate-modified allele specific primer extension mediated by Pfu polymerase identified both homozygous for *SLC26A4* gene c.919-2A > G variant in two patients clinically diagnosed as LVAS by temporal bone CT scan. The genetic results from this method are consistent with that of DNA sequencing. The data suggest that exo^+^ polymerase-mediated 3′ terminal phosphorothioate-modified primer extension is reliable in the identification of *SLC26A4* gene high-frequency variant prior to high-resolution CT scan. The method is extremely suitable for quickly molecular etiologic screening and early diagnosis and aggressive prevention therapy of LVAS.

## Introduction

Large vestibular aqueduct syndrome (LVAS) is an autosomal recessive genetic hearing loss disorder with a high incidence which is mainly accompanied by progressive and fluctuating hearing loss (Tong et al. 1997; Claros et al. 2017). It is well known that the solute carrier family 26, member 4 (*SLC26A4*) gene plays a critical role in the development of LVAS, and about 90% of LVAS is closely attributed to *SLC26A4* gene variants (Nishio et al. 2016; Chao et al. 2019; Kim et al. 2019). The *human SLC26A4* gene is mapped to chromosome 7q31, encompasses 21 exons and contains a 2343 bp open reading frame encoding a protein of 780 amino acids. To date, more than 100 of *SLC26A4* gene variants have been identified and described as causally related to hereditary hearing impairment (https://hereditaryhearingloss.org/).

Before the clinical application of gene diagnosis, the imageological examination was the only way of LVAS diagnosis and had stated clearly that LVAS is the most common inner ear malformation (Connor et al. 2019; Yang and Liu 2019).

Previous studies revealed that *SLC26A4* gene variant has obvious racial specificity, LVAS patients from different races with unique variant spectra and different variant frequencies (Berrettini et al. 2005; Hu et al. 2007; Lee et al. 2008). For years, the interest in *SLC26A4* gene variant closely associated with Chinese LVAS individuals has been focused on c.919-2A > G and c. 2168A > G variants (Hu et al. 2007; Zhu et al. 2012), The two most common types (c.919-2A > G and c. 2168A > G) accounted for 69.1% (Hu et al. 2007) and 33.06% (Guo et al. 2008) variants respectively. Therefore, screening the high-frequency variant can early diagnose LVAS patients and find variant carriers and then taking measures to prevent further hearing loss by keeping LVAS patients from cold and head injury.

We have previously reported that exo^+^ polymerase-mediated 3′ terminal exonuclease-resistant phosphorothioate-modified allele specific primer extension formed a molecular switch sensitive to single nucleotide discrimination (Zhang and Li 2003; Zhang et al. 2005). For 3′ allele-specific primers with phosphorothioate-modification, perfect-matched primer turns on and mismatched primer turns off DNA polymerization. Exo^+^ polymerases generated products only from perfect-matched primers and no products from mismatched primers, which provided an unambiguous readout for yes or no in a specific variant detection assay. In this study, we will construct a double PCR technology platform mediated by variant sensitive molecular switch for rapid screening the *SLC26A4* gene c.919-2A > G and c.2168A > G high-frequency variants and explore strategies for clinical genetic diagnosis.

## Materials and methods

### Reagents

Pfu DNA polymerase with strong 3′ → 5′ exonuclease activity was purchased from TaKaRa Bio Inc. (Dalian, China). KOD-Plus-Mutagenesis Kit used in this study was obtained from TOYOBO Inc. (Shanghai, China). pMD19-T vector was the product of TaKaRa Bio Inc. (Dalian, China). Both unmodified primers and phosphorothioate-modified primers were synthesized commercially by TaKaRa Bio Inc. (Dalian, China).

### Primer design

There are three types of primers: wild template preparation primers, mutagenesis primers and detection primers. The unmodified primers for wild type *SLC26A4* gene preparation were designed based on the information of the Genebank database (NC_000007.14). The mutagenesis primers for *SLC26A4* gene c.919-2A > G and c.2168A > G variants through inverse PCR were designed to create point variants in the corresponding sites according to the Muta Primer 2.0 software. Allelic specific detection primers targeting wild and mutant templates were both designed with 3′ terminal phosphorothioate-modification. The 3′ terminal upstream to the -2 base (T or C) of the reverse primer of c.919-2A > G and the forward primer of c.2168A > G (A or G) were the respective variant loci. All primers were synthesized by TaKaRa Bio Inc according to the following sequences as illustrated in Table 1, and the graphic illustration of primers is shown as Additional file 1.

**Table 1:**
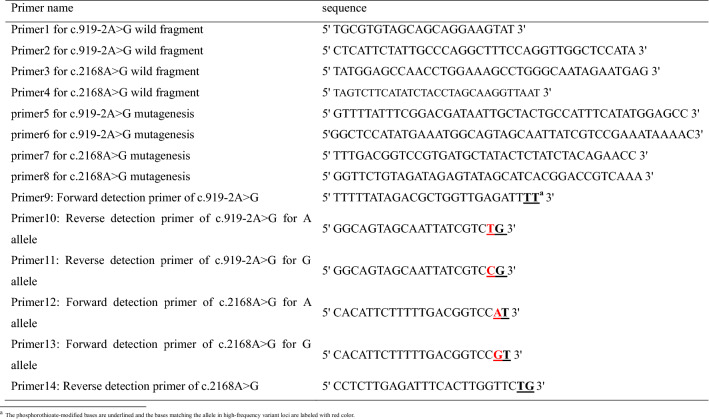
Sequences of all the primers used in this study

### Vector construction and identification

Overlapping PCR generated *SLC26A4* gene fragment harboring c.919-2A > G and c.2168A > G flanking sequence, which was subsequently added A base and then inserted into the pMD19-T vector followed by transforming into *E.coli JM109* competent cells. After 37℃ incubation for 12 h, some clones were formed on the LB-agar plates containing X-Gal, IPTG and Ampicillin. White clones were randomly picked up for bacilli PCR amplification reaction with the M13 primers as an initial identification of the inserted DNA overlapped fragment by their size, and 1.0 ml bacilli of each positive clone were sent to Invitrogen Biotechnology Co. Ltd. (Shanghai, China) for DNA sequencing.

Inverse PCR according to the manufacturer’s protocol was performed twice for site-directed mutagenesis of c.919-2A > G and c.2168A > G variant in *SLC26A4* gene. Following the degradation of the methylated DNA template by *DpnI* endonuclease, the mutant circular PCR product was transformed into *E.coli JM109* competent cells again. Similarly, the *SLC26A4* gene c.919-2A > G and c.2168A > G mutant template was also confirmed by sequence analysis.

### Extension of phosphorothioate-modified primers by exo^+^ polymerase

Two-directional primer extension was setup. Following denaturation at 95 °C for 2 min, the primer extension was carried out for 30 cycles as follows: 30 s for denaturation at 95 °C, 30 s for annealing at 60 °C, and 20 s for extension at 72 °C. After the 30 cycles, an extra extension of 2 min was done before the reaction mixture was cooled down to 4 °C. The primer extension reaction was performed in a total volume of 30 µL with 20 pg of template, 0.2 mmol/L dNTP, 10 Ku/L of polymerase, 10 pmol/µL of both sense and antisense primers, and 2.5µL of the 10 × Pfu polymerase reaction buffer which provides a final concentration of 10 mmol/L KCl, 20 mmol/L Tris–HCl (pH 8.8 at 25 °C), 10 mmol/L (NH_4_)_2_SO_4_, 2 mmol/L MgSO_4_, 0.1% Triton X-100. Electrophoresis in a 2.0% agarose EtBr gel at 8 V/cm in 0.5 × TBE buffer was used to check whether the two-directional primer extension products were produced or not.

### High-frequency variants screening on clinical LVAS patient

2.0 ml of peripheral vein blood was obtained from two cases from the second affiliated hospital of University of South China, who were diagnosed as LVAS by the high-resolution temporal bone CT imageological examination, and the *human* genomic DNA from the peripheral blood lymphocytes was extracted using phenol–chloroform extraction method. Pfu DNA polymerase combining with 3′ terminal phosphorothioate-modified primers targeting c.919-2A > G and c.2168A > G variants were utilized to screen the *SLC26A4* gene on LVAS patients whether harboring either c.919-2A > G or c.2168A > G high-frequency variant. Furthermore, another PCR amplification was also carried out to separately get exon7 + 8 and exon19 fragment in *SLC26A4* gene, which is suitable product including c.919-2A > G and c.2168A > G locus, and 1.0 ml of the purified primer extended products were further used for sequence analysis from Invitrogen Biotechnology Co. Ltd. (Shanghai, China).

## Results

### Construction and identification of vector

**Fig. 1:**
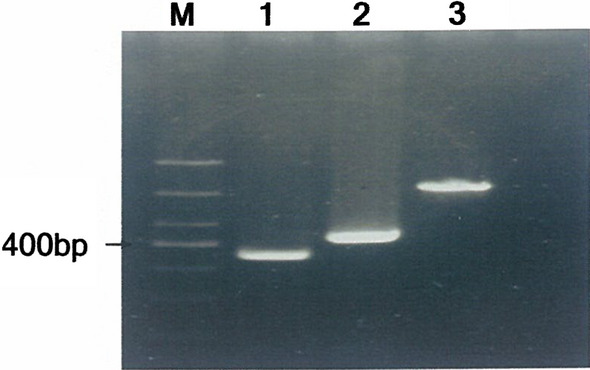
DNA product from Overlapping PCR. M: 100 bp DNA marker; 1: PCR product harboring *SLC26A4* gene c.919-2A > G flanking sequence, and the DNA fragment is 360 bp; 2: PCR product harboring *SLC26A4* gene c.2168A > G flanking sequence, and the DNA fragment is 444 bp; 3: overlapping PCR products using the product 1 and 2 as temples, and the DNA fragment is 766 bp

**Fig. 2:**
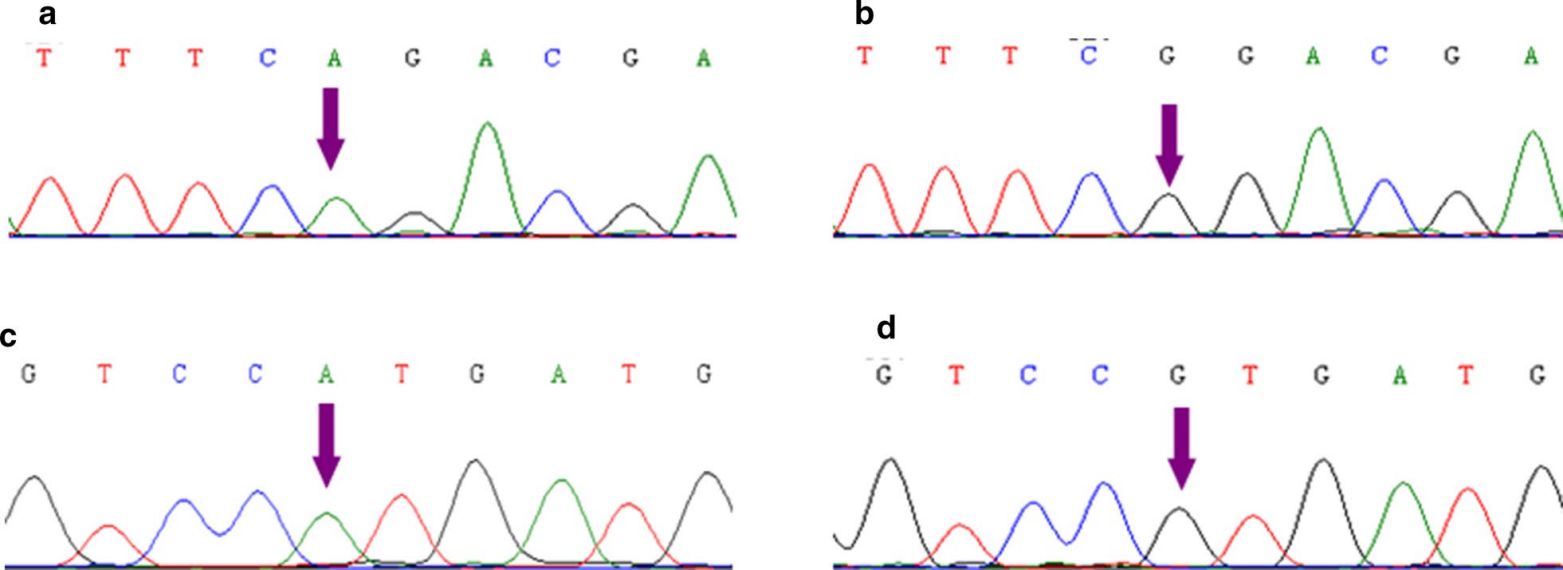
Representative of DNA sequencing of the constructed vector. **a** DNA sequencing result residing c.919-2A > G site in *SLC26A4* gene for A base; **b** DNA sequencing result residing c.919-2A > G site in *SLC26A4* gene for G base; **c** DNA sequencing result residing c.2168A > G site in *SLC26A4* gene for A base; **d** DNA sequencing result residing c.2168A > G site in *SLC26A4* gene for G base

PCR-generated wild DNA fragment separately harboring *SLC26A4* gene c.919-2A > G and c.2168A > G flanking sequence were successfully overlapped (Fig. 1). After the overlapped DNA fragment was inserted into the pMD19-T vector and subsequently transformed into *E.coli JM109* competent cells, some white clones were formed on the X-Gal/IPTG/ampicillin/LB-agar plates and randomly picked up for bacilli PCR amplification with the M13 primers to obtain positive clones. DNA sequencing not only confirmed the correctness of wild vector sequence as wild type template (Fig. 2a, c), and also documented the mutagenesis of c.919-2A > G and c.2168A > G through inverse PCR: the existence of *SLC26A4* gene c.919-2G and c.2168G variants in the mutant vector as mutant type template (Fig. 2b, d).

### Nucleotide discrimination by exo^+^ polymerase

**Fig. 3:**
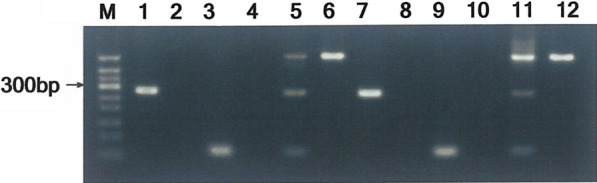
Discrimination of *SLC26A4* gene c.919-2A > G and c.2168A > G variants by Pfu DNA polymerase-mediated phosphorothioate-modified primer extension. M is 50 bp DNA marker; Lanes 1 and 8 is specific primer extension products from the primer of c.919-2A > G site for A allele, Lanes 2 and 7 are specific primer extension products from the primer of c.919-2A > G site for G allele, Lanes 3 and 10 are specific primer extension products from the primer of c.2168A > G site for A allele, Lanes 4 and 9 are specific primer extension products from the primer of c.2168A > G site for G allele, Lanes 5 and 12 are specific primer extension products from primer mixture of c.919-2A > G site for A allele and c.2168A > G site for A allele, Lanes 6 and 11 are specific primer extension products from primer mixture of c.919-2A > G site for G allele and c.2168A > G site for G allele; Lanes 1 to 6 are the PCR products using wild vector as wild template; Lanes 7 to 12 are the PCR products using mutant vector as mutant template. The specific DNA product targeting c.919-2A > G and c.2168A > G loci is 280 bp and 65 bp respectively. Lanes 5, 6, 11 and 12 generate an extra 518 bp nonspecific fragment from a pair of amplification primers consisting of the forward detection primer of c.919-2A > G and the reverse detection primer of c.2168A > G

The data illustrated in Table 2 and Fig. 3 clearly showed that c.919-2A > G and c.2168A > G allelic specific primers perfectly matching wild type allele template were extended while no products were produced from point-mutated LVAS-associated mutant type allele template, and also allelic specific primers perfectly matching point-mutated LVAS-associated mutant type allele template were extended but no products were observed from wild type allele template. Furthermore, we carried out double PCR for *SLC26A4* gene c.919-2A > G and c.2168A > G variants identification, and similarly, the matched primer extension was extended while the mismatched primer was not. But it was important to remind that duplex PCR generated an additional 518 bp nonspecific fragment regardless of primers perfectly matching constructed vector template or not, which was the product from the pair of amplification primer consisting of the forward detection primer of c.919-2A > G and the reverse detection primer of c.2168A > G. Since 3′ → 5′ exonuclease activity of high-fidelity DNA polymerase can efficiently detect and proofread the mismatched base of the allele specific primer, once high-fidelity DNA polymerase combining with 3′ terminal exonuclease-resistant phosphorothioate-modified allele specific primer, the perfect-matched primer turned on and the mismatched primer turned off DNA polymerization. That provides an unambiguous readout for yes or no in single nucleotide discrimination: only the perfect-matched phosphorothioate-modified primer extension mediated by pfu DNA polymerase generated specific products and the mismatched primers extension not.

**Table 2:** Discrimination of c.919-2A > G and c.2168A > G mutations in *SLC26A4* gene through the combination of 3′ phosphorothioate-modified primers and Pfu DNA polymerase

Lane	Template	Mutation locus	Primer ( 3′base-pairing with template)	Product (Yes or No)
1	Wild template	c.919-2A > G	c.919-2A allele (matched)	Yes
2	Wild template	c.919-2A > G	c.919-2G allele (mismatched)	No
3	Wild template	c.2168A > G	c.2168A allele (matched)	Yes
4	Wild template	c.2168A > G	c.2168G allele (mismatched)	No
5	Wild template	c.919-2A > G + c.2168A > G	c.919-2A allele + c.2168A allele (matched)	Yes
6	Wild template	c.919-2A > G + c.2168A > G	c.919-2G allele + c.2168G allele (mismatched)	No
7	Mutant template	c.919-2A > G	c.919-2G allele (matched)	Yes
8	Mutant template	c.919-2A > G	c.919-2A allele (mismatched)	No
9	Mutant template	c.2168A > G	c.2168G allele (matched)	Yes
10	Mutant template	c.2168A > G	c.2168A allele (mismatched)	No
11	Mutant template	c.919-2A > G + c.2168A > G	c.919-2G allele + c.2168G allele (matched)	Yes
12	Mutant template	c.919-2A > G + c.2168A > G	c.919-2A allele + c.2168A allele (mismatched)	No

### Clinical application on screening the *SLC26A4* high-frequency variant

**Fig. 4:**
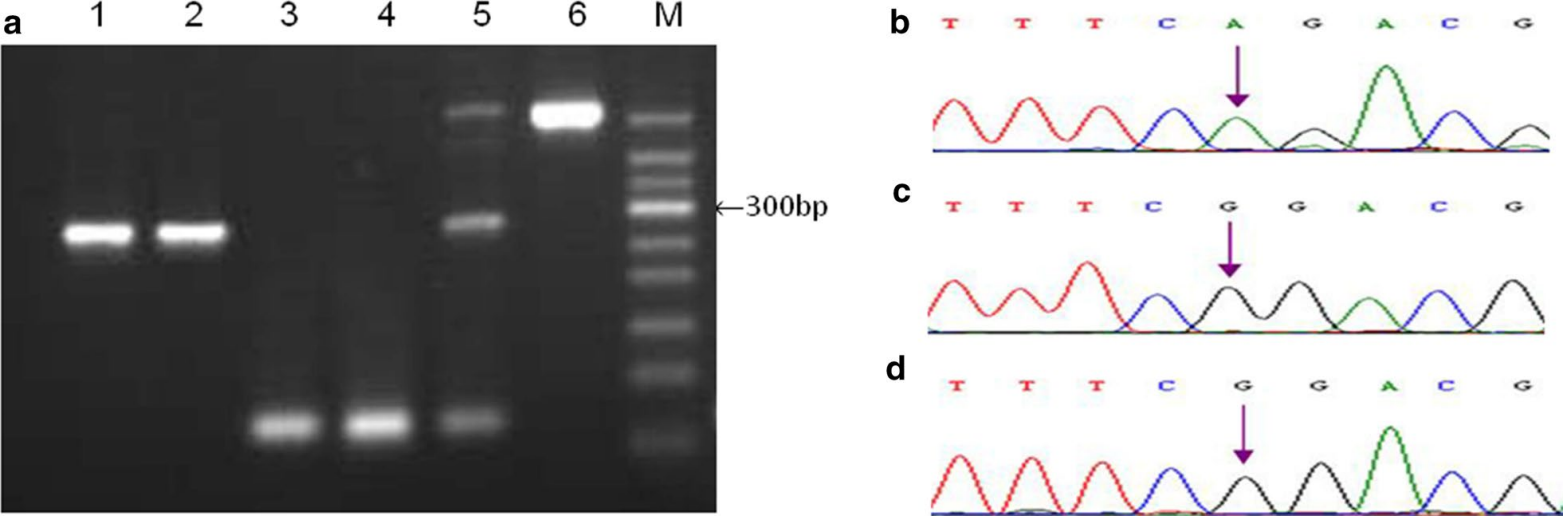
The clinical application of Pfu DNA polymerase-mediated 3′ terminal phosphorothioate-modified primer extension on screening the *SLC26A4* high-frequency variant. **a** M is 50 bp DNA marker; Lanes 1, 2, 5 and 6 are specific primer extension products from primer mixtures of c.919-2A > G site for G allele and c.2168A > G site for G allele, which perfectly match mutant template; Lanes 3 and 4 are specific primer extension products from primer mixtures of c.919-2A > G site for A allele and c.2168A > G site for A allele, which perfectly matches wild template. Lanes 1 and 3 represent LVAS case1, and Lanes 2 and 4 represent LVAS case2. Lanes 5 is mutant vector as the positive control; Lanes 6 is wild vector as the negative control. **b**, **c** and **d** Sequence analysis of *SLC26A4* gene. **b** Represents healthy volunteer, **c** and **d** respectively represent LVAS case1 and 2

Pfu DNA polymerase-mediated phosphorothioate-modified primer extension was used to identify the *SLC26A4* gene variant on the LVAS patients who had been diagnosed as LVAS by temporal bone CT scan. The two LVAS patients both harbored c.919-2A > G variant and were both homozygous for c.919-2A > G variant confirmed by DNA sequencing (Fig. 4).

## Discussion

LVAS is frequently seen in children and teenagers, and congenital or acquired sensorineural hearing loss is its main manifestation. Now high-resolution temporal bone CT and MRI imageological examination are still the gold standards and a prerequisite for the correct diagnosis of LVAS. However, most domestic hospitals not only lack the requirement suitable for imageological diagnosis but also have no technologies that seem appropriate. As a result, most LVAS patients cannot receive timely correct diagnosis so that the doctor cannot provide effective treatment and prevention advice to him. Thankfully, with the clinical application of deafness-associated gene variant screening, it has demonstrated the particular advantages in the etiologic diagnosis of deafness.

Numerous studies indicate that c.919-2A > G and c.2168A > G variants in *SLC26A4* gene are the high-frequency variants associated with Chinese LVAS individuals(Hu et al. 2007; Guo et al. 2008; Jiang et al. 2010; Li et al. 2011), which is similar to other patients in East Asia(Shin et al. 2012; Tsukamoto et al. 2003) and benefits *SLC26A4* gene high-frequency variant screening in clinical application. Remarkably, none can effectively cure LVAS patients from etiology to symptoms until now, so early diagnosis and aggressive prevention therapy have great practical significance in preventing hearing further decline. Detecting LVAS-associated high-frequency variant c.919-2A > G and c.2168A > G is extremely useful in the early diagnosis and intervention of LVAS, and the current research has focused on how to quickly screen *SLC26A4* gene variant for foundationally preventing the occurrence of LVAS. Prohibiting either c.919-2A > G or c.2168A > G variant carrier from marrying another one with the same variant is thus the most effective way to protect the occurrence of LVAS.

As we all know, many gene variants assay technologies have been developed over the past decades, and the most popular methods are different variants of allele-specific primer extension mediated by DNA polymerases: High-throughput sequencing, Sanger’s method, restriction fragment length polymorphism (RFLP), multiplex ligation-dependent probe amplification (MLPA) and so on. High-throughput sequencing is a revolutionary technological innovation in gene sequencing researches and also known as next-generation sequencing (NGS). Although NGS is characterized by low cost and high-throughput data making it widely applied in multi-level researches, a high rate of false positives and requirement for prior PCR amplification or demands for expensive equipment remain the major obstacles to their effective wide applications. Sanger’s method is also known as the “chain-termination method” and still considered the gold standard of gene sequencing technology today since it provides a high degree of accuracy and long-read capabilities, but the low throughput and high cost of sample preparation make it difficult to support a diverse range of applications in many research areas. RFLP is based on the alterations in restriction sites, so it is not suitable to assay gene variants not residing within restriction sites. MLPA relies on sequence-specific probe hybridization of genomic DNA, followed by multiplex-PCR amplification of the hybridized probe, so a suitable DNA extraction method must be chosen to keep DNA integrity and purity. Undoubtedly, exo + polymerase-mediated phosphorothioate-modified primer extension bypasses the limitation of template-dependent product generation by exo + polymerases through exonuclease-digestible allele-specific primers extension. Generally, it is easy to operate, economic and reliable, and these advantageous features make it very suitable for clinical genetic detection and variant screening. So in this study, we applied the variant sensitive molecular switch consisting of 3′ terminal exonuclease-resistant phosphorothioate-modified allele specific primer combining exo^+^ polymerase in single-base discrimination of c.919-2A > G and c.2168A > G variants in *SLC26A4* gene.

In our present study, the data from DNA sequence analysis proved that large amounts of wild and mutant type templates were successfully prepared by inserting the *SLC26A4* gene overlapped PCR products into the pMD19-T vector and inverse PCR respectively. The two-directional primer extension showed that exo^+^ polymerases, combining 3′ terminal phosphorothioate-modified mismatched primer, work as an off-switch in DNA polymerization, the perfect match primer turned on and the mismatched primer turned off DNA polymerization respectively. So only allelic specific primer perfectly matching template yielded specific PCR product while allelic specific primer mismatching template not, which provided an unambiguous readout for yes or no in single nucleotide discrimination. The consequence of the off-switch effect resulted from the exonuclease-resistant property of the phosphorothioate-modification that blocked mismatched base excision of the primer during 3′ → 5′ exonuclease proofreading procedure. Remarkably, an extra 518 bp nonspecific fragment was generated from the constructed vector template regardless of primers perfectly matching or not, while no nonspecific fragment was produced from the genomic DNA. The extra 518 bp nonspecific fragment was the product from the pair of amplification primer consisting of the forward detection primer of c.919-2A > G and the reverse detection primer of c.2168A > G. Of course, owing to ineffective amplification when using genomic DNA as the PCR template, the additional 518 bp nonspecific product was not yielded. So when we applied Pfu DNA polymerase-mediated phosphorothioate-modified primer extension to screening the *SLC26A4* gene on LVAS patients diagnosed as LVAS by temporal bone CT scan whether harboring c.919-2A > G or c.2168A > G variant, we could only observe specific PCR product without 518 bp nonspecific fragment. Actually, when using primer mixtures of c.919-2A > G site for G allele and c.2168A > G site for G allele, only specific primer of c.919-2A > G site for G allele yielded specific PCR product, which showed that the two LVAS patients both harbored c.919-2A > G variant. For further identifying the allele type of LVAS patients, the double PCR from primer mixtures of c.919-2A > G site for A allele and c.2168A > G site for A allele were carried out again. On the contrary, not specific primer of c.919-2A > G site for A allele but c.2168A > G site for A allele yielded specific PCR products. Thus it showed that both LVAS cases were homozygous for c.919-2A > G variant. Moreover, DNA sequencing also confirmed the reliability of exo^+^ polymerase-mediated phosphorothioate-modified primer extension in the identification *SLC26A4* c.919-2A > G and c.2168A > G variant prior to high-resolution CT scan. The method is extremely suitable for quickly molecular etiologic screening. As an important testing means, genetic diagnosis of c.919-2A > G and c.2168A > G variants and imageological examination are complementary with each other and can improve early diagnosis and deepen aggressive prevention therapy of LVAS.

Therefore, an easy, cheap and fast molecular diagnostics of c.919-2A > G and c.2168A > G variants in *SLC26A4* gene will greatly benefit patients. Through the wide application in clinical otology, genetic diagnostic techniques can guide pre-lingual deaf patients as soon as possible to make use of residual hearing with hearing aids or implanted electronic cochlea for preventing variable dumb and avoiding any further damage to the hearing of deafness patients themselves; More importantly, it can also be used for the assessment of the risk of post-lingual deaf patients, prenatal diagnosis or prognosis, and patients with the marriage, birth genetic information to prevent the occurrence of LVAS patient fundamentally.

## Supplementary information

**Additional file 1. **Graphic illustration of primer sequences.

## Data Availability

The datasets used and/or analyzed during the current study are available from the corresponding author on reasonable request.
