# Supplementary material for: A novel genotyping technique for discriminating LVAS-associated high-frequency variants in SLC26A4 gene
Source: AMB Express. 2020 Sep 15;10:166. doi: 10.1186/s13568-020-01102-7 (PMC7492351; doi:10.1186/s13568-020-01102-7)
Supplement: Supplementary file 1 — Additional file 1. Graphic illustration of primer sequences. [file 13568_2020_1102_MOESM1_ESM.doc]

**
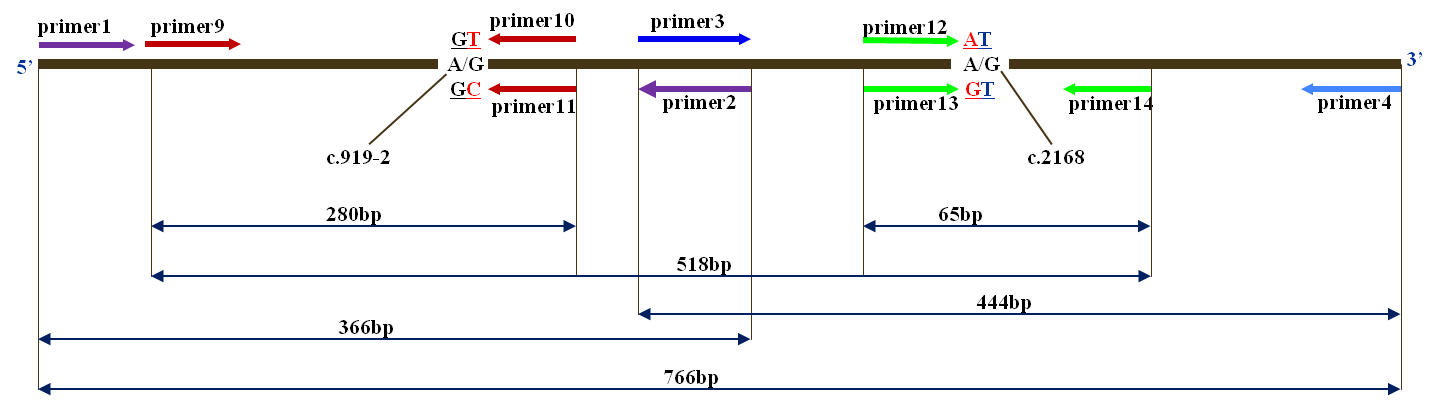
**

Graphic illustration of primer sequences. Primer1+Primer2 are for generating 360bp wild fragment of c.919-2A>G variant, Primer3+Primer4 are for generating 444bp wild fragment of c.2168A>G variant; Primer9+Primer10 are for detecting c.919-2A>G variant for A allele with a 280bp fragment; Primer9+Primer11 are for detecting c.919-2A>G variant for G allele with a 280bp fragment; Primer12+Primer14 are for detecting c.2168A>G variant for A allele with a 65bp fragment, Primer13+Primer14 are for detecting c.2168A>G variant for G allele with a 65bp fragment; Primer9+Primer14 can produce an extra 518bp nonspecific fragment from the constructed vector template while no nonspecific fragment can be produced from the case’s genomic DNA; Primer2 and primer3 are completely complimentary for generating *SLC26A4* gene fragment harboring c.919-2A>G and c.2168A>G flanking sequence by overlapping PCR. The 3’ terminal upstream to the -2 base of primer10(T) and primer11(C) is targeting c.919-2A>G variant locus, and primer12(A) and primer13(G) is targeting c.2168A>G variant locus.
